# Evaluation of Chronic Lateral Ankle Instability With an Ankle Sprain Simulator: A Controlled Study in Physically Active Subjects

**DOI:** 10.1002/jfa2.70182

**Published:** 2026-07-15

**Authors:** Matthieu Lalevée, Marie‐Anne Melone, Maxime l’Hermette, Julien Beldame, Cesar de Cesar Netto, Eli Schmidt, Jason Wilken

**Affiliations:** ^1^ CETAPS—UR 3832 Research Center for Sports and Athletic Activities Transformations University of Rouen Mont Saint Aignan Normandy France; ^2^ Department of Orthopedic Surgery Rouen University Hospital Rouen France; ^3^ Carver College of Medicine Department of Orthopedics and Rehabilitation University of Iowa Iowa Iowa USA; ^4^ Department of Pulmonary Thoracic Oncology and Respiratory Intensive Care Rouen University Hospital Univ Rouen Rouen France; ^5^ Institut Clinique du Pied Ramsay Santé Clinique Blomet Paris France; ^6^ Department of Orthopedic Surgery Duke University Durham North Carolina USA; ^7^ Department of Rehabilitation Medicine University of Minnesota Minneapolis Minnesota USA; ^8^ Department of Physical Therapy and Rehabilitation Science University of Iowa Iowa Iowa USA

**Keywords:** ankle inversion, ankle kinematics, ATFL, CFL, instability, inversion velocity

## Abstract

**Purpose:**

Chronic lateral ankle instability (CLAI), categorized by repetitive ankle sprains, has previously been studied utilizing either questionnaires or ankle sprain simulators. However, a quantitative tool that safely creates an unexpected ankle sprain during gait has not been proposed. This study aimed to determine if a novel ankle sprain simulator differentiates between CLAI and healthy controls and compare findings to known measures of impaired function.

**Methods:**

Thirty‐two feet were assigned to the CLAI group or the control group. Participants walked on a treadmill wearing boots designed to induce unexpected ankle inversion. To do so, the device was triggered randomly during swing to induce instability during loading response. Maximal inversion velocities (MIV) were measured immediately after initial contact. A Ratio MIV was determined by dividing the MIV of the simulated sprain by the average MIV of the 5 steps preceding the simulated sprain. Also, Identification of Functional Ankle Instability (IdFAI) and the Foot Ankle Ability Measurement (FAAM) questionnaires were collected.

**Results:**

Mean MIV in the CLAI and Control groups were 213.5 ± 54.7°/s and 177.0 ± 64.2°/s (*p* = 0.02), while mean Ratio MIV were 1.22 ± 0.13 and 1.08 ± 0.08 (*p* < 0.001), respectively. The area under the curve to predict CLAI was 0.83 (95% CI; [0.73; 0.93]) for Ratio MIV with a value greater than 1.15 showing 68% sensitivity and 93.8% specificity. Ratio MIV was moderately correlated with IdFAI (*ρ* = 0.50; *p* < 0.0001) and weakly correlated with impairments in activities of daily living (*ρ* = −0.45; *p* < 0.001) and sports (*ρ* = −0.43; *p* < 0.001).

**Conclusion:**

Inversion velocities caused by the ankle sprain simulator differentiated CLAI from healthy controls. Ratio MIV was associated with measurements of functional CLAI, impairments in ADLs and sports.

## 1 Introduction

Lateral ankle sprains (LAS) are one of the most common musculoskeletal injuries and leads to a significant socioeconomic burden [1, 2]. Although commonly considered as a benign injury, up to 40% of subjects who experience one lateral ankle sprain (LAS) will develop chronic lateral ankle instability (CLAI) [3, 4]. Those who develop CLAI are at an increased risk of developing both short‐ and long‐term sequalae, including osteochondral lesions [5] and ultimately post‐traumatic ankle osteoarthritis [6, 7].

Despite this, paradoxically the management of LAS and CLAI has not significantly changed over the years [8, 9]. A major factor impeding progress in CLAI prevention is its multifaceted nature, with providers of varied specialties working to address ankle instability from their unique viewpoints [10]. One example of this is the frequent distinction between functional and mechanical instability in the literature [11, 12]. Physiatrists, athletic trainers, and physiotherapists often focus on the functional components of instability such as self‐reported feelings of the ankle “giving way”, changes in muscle activation [13], and alterations in balance [14, 15] while orthopedic surgeons commonly focus on mechanical components of instability such as ligamentous laxity [16, 17, 18].

Additionally, there is a lack of tools to quantify, and therefore objectively diagnose CLAI [19]. Questionnaires are commonly used to identify factors that predict CLAI and assess outcomes. Donahue et al. combined the Cumberland Ankle Instability Tool (CAIT) and the Ankle Instability Instrument to predict ankle instability status [20]. The resulting measure is called the Identification of Functional Ankle Instability (IdFAI) questionnaire [21]. However, this questionnaire focuses on the functional aspects of CLAI (i.e., the perceived symptoms of the ankle giving way). Despite the IdFAI's focus on the functional component, more recent models of CLAI stress the importance of the combination of both functional and mechanical CLAI [10]. Additional models seek to account for the cumulative effect of the interplay between these factors [22].

Ankle sprain simulators may help overcome limitations associated with reliance on questionnaires and patient self‐reported history of ankle sprains [23, 24]. By assessing CLAI quantitatively, a simulator can assess the ankle during the moment of instability, thereby holistically assessing all factors (functional and mechanical) that cause an ankle to sprain. Sprain simulators have previously been used to differentiate CLAI subjects from controls using maximum ankle inversion velocity during a simulated inversion trauma [25, 26]. While the potential to provide quantitative data to identify CLAI is promising, these previous ankle sprain simulators have only evaluated CLAI in either a pseudostatic position [27, 28] or during landing and in a way that was expected or predictable by the participant [29, 30, 31, 32]. Using a supination sprain simulator, Chu et al. reported maximal inversion velocities of 114°–203°/s during simulated sprains, compared with only 22°–85°/s during common sporting movements [25]. For these sprain simulators, a risk threshold of 300°/s was proposed, based on a real injury event reaching 632°/s [25]. However, most of these earlier paradigms tested the ankle in a static position or during a landing task, not during walking [23]. Moreover, when the perturbation is expected rather than unexpected, the protective motor response of the ankle is altered [31]. By delivering an unexpected perturbation during gait, the present device aims to overcome these limitations and to reproduce more closely the conditions of a real lateral ankle sprain.

Therefore, we conducted a study that primarily aimed to differentiate CLAI subjects from controls using a novel ankle sprain simulator triggered unexpectedly while walking. We also aimed to determine the association between sprain simulator results and impairments of daily living and impairments in sports.

## 2 Materials and Methods

### 2.1 Study Design

This study received institutional review board approval (IRB 202202219) and complied with the Health Insurance Portability and Accountability Act (HIPAA). Participants received detailed information regarding the protocol and signed an informed consent form prior to participation. The STrengthening the Reporting of OBservational studies in Epidemiology (STROBE) recommendations were followed [33, 34].

### 2.2 Population

Recruitment emails were sent to introduce the study and ask for physically active volunteers. According to Lin et al., the physically active population includes athletes, military personnel, or active subjects under the age of forty [35]. Both healthy volunteers as well as individuals with suspected CLAI were invited to participate in this study. Volunteers younger than 18 years, heavier than 95 kg (209 lbs), those with an history of ankle sprain in the last 3 months, those with neurological or sensorimotor impairment, osteoporosis, or any active lower extremity disorder were not considered.

To determine the group in which the ankles were included, the International Ankle Consortium recommended selection criteria for CLAI patients in controlled research was used [1] and is as follows:

— The CLAI group included ankles with at least one LAS whose first episode occurred more than 12 months before the start of the study AND
  ◦ At least two or more ankle sprains to the same ankle OR
  ◦ At least two episodes of the ankle giving way in the 6 months before the study OR
  ◦ A feeling of ankle instability confirmed by an IdFAI score greater than 10.
— The Control group included ankles without any history of LAS, giving way, or feeling of ankle instability on questionnaires.

Ankles that did not meet these criteria or were the contralateral ankle in cases of unilateral CLAI, were excluded from analysis.

### 2.3 Study Protocol

**FIGURE 1:**
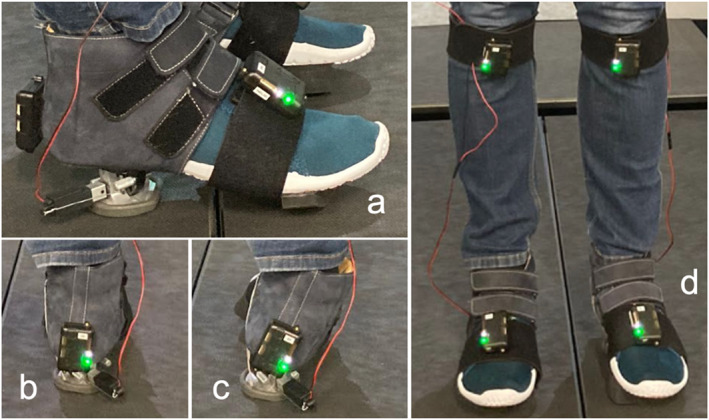
Instability boots; (a) Lateral view of right foot; (b) Posterior view of right foot with boot in stable position; (c) Posterior view of right foot with boot in unstable position; (d) Frontal view.

The sprain simulator (Myolux Athletik) used was developed and previously validated for CLAI rehabilitation [36]. This device, which moves around the Henke axis, was a prototype designed for the study. It consists of a boot that surrounds the foot and ankle, ensuring stability of the ankle joint and preventing excessive motion and injury (Figure 1).

Gait analysis was performed using the Computer Assisted Rehabilitation Environment system (CAREN) virtual reality treadmill (Motek, Amsterdam, Netherlands) by having participants walk on the treadmill wearing the sprain simulator and secured by a suspension harness to prevent falls (Supporting Information S1: Figure SA).

Prior to gait analysis, each subject completed the IdFAI and the Foot Ankle Ability Measurement (FAAM) for each ankle [21, 37]. Participant age, BMI, sex, and sport practice information (type of sport, sport for leisure or competition, and mean hours of sport per month) were also collected.

During an initial accommodation period the sprain simulators were locked in a stable position while the subject accommodated to the environment and study setup (Figure 1). After at least 3 min of walking, a member of the research team disengaged the locking mechanism via remote control at random during swing phase. This allowed for an unexpected instability moment during the subsequent loading phase, where the participants ankle was allowed to invert naturally. In this manner, surprise moment of ankle instability was reliably and reproducibly created, mimicking a real‐life ankle sprain.

The sprain simulator was coupled with Inertial Moment Unit (IMU) sensors (Isen 3.0 STT Systems) (Figure 1). Data from these sensors were collected through the iSen software interface. The inversion angles and velocities during the first stance phase after the triggering of instability were analyzed.

Finally, all subjects were video recorded from the back. This video was synchronized with the gait analysis and IMU sensors to improve data interpretation (Supporting Information S1: Figure SA).

### 2.4 Assessment Criteria

**FIGURE 2:**
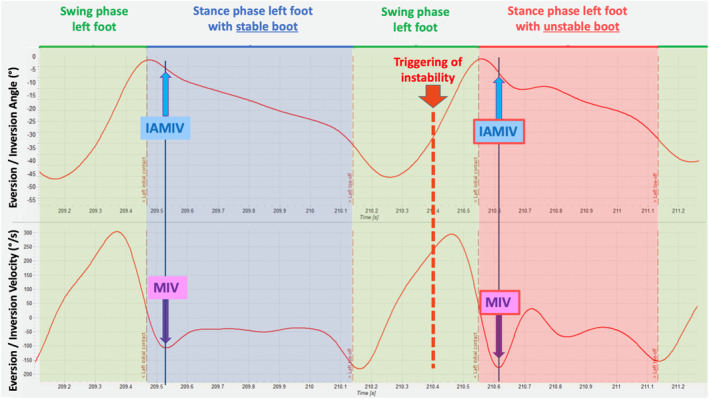
Eversion/Inversion angle and velocity curves during the onset of ankle instability. *Inversion represented by values less than 0, eversion by values greater than 0*. IAMIV, Inversion Angle at MIV Time; MIV, Maximal Inversion Velocity.

Maximal inversion velocity (MIV), measured in degrees per second, was defined as the greatest velocity the ankle experiences in inversion during the simulated ankle sprain moment. This measure has been previously determined to be an accurate parameter to assess CLAI, as CLAI patients have been shown to demonstrate a significantly greater MIV [25, 26]. MIV was measured during the loading phase, between the heal strike and the midstance phase (Figure 2). The inversion angle at the moment of MIV (IAMIV) was also collected to quantify the angular distance the ankle traveled in inversion before the subject was able to start counteracting and decrease the inversion velocity (Figure 2). Throughout this study, inversion is expressed as negative values and eversion as positive values. The maximal inversion velocity (MIV) and the inversion angle at maximal inversion velocity (IAMIV) are reported as absolute magnitudes.

Due to high intersubject variability for both MIV and IAMIV, values were normalized for each individual. The value of the simulated trauma was divided by the average value of the five cycles preceding the simulated sprain, defining the Ratio MIV and Ratio IAMIV, respectively (Supporting Information S1: Figure SB). Equations for calculating Ratio MIV and Ratio IAMIV are as follows:

$$\text{Ratio}\;\text{MIV}=\frac{\text{MIV}\;\left(\text{simulated}\;\text{trauma}\right)}{\left(-5\text{MIV}+-4\text{MIV}+-3\text{MIV}+-2\text{MIV}+-1\text{MIV}\right)/5}$$

$$\text{Ratio}\;\text{IAMIV}=\frac{\text{IAMIV}\;\left(\text{simulated}\;\text{trauma}\right)}{\left(-5\text{IAMIV}+-4\text{IAMIV}+-3\text{IAMIV}+-2\text{IAMIV}+-1\text{IAMIV}\right)/5}$$

### 2.5 Statistical Analysis

Normality and heteroskedasticity of data were assessed with the Shapiro‐Wilk test and the Levene's test. The groups were compared using Student *t*–test for normal and Mann–Whitney *U*‐test for nonnormal variables. Percent of each sex and sport characteristics between groups were compared using a 2‐tailed Fisher exact test. Receiver‐Operating‐Characteristic curves were performed to assess the ability to predict CLAI according to the explanatory variables. The area under the curve and 95% confidence intervals were calculated. The Youden's index was used to define diagnostic thresholds.

Spearman's correlations were used to determine the linear association between the explanatory variables and the IdFAI score, the FAAM Activities of Daily Living Subscale and the FAAM Sports Subscale. Correlations were judged to be very strong from 1 to 0.9, strong from 0.9 to 0.7, moderate from 0.7 to 0.5, low from 0.5 to 0.3 and poor from 0.3 to 0. Because some participants contributed both ankles, the main analyses treated each ankle as an observation. To check that within‐subject correlation did not bias the results, we performed a sensitivity analysis at the participant level as follows: for participants with two included ankles, their two values were averaged to obtain a single value per participant, and the between‐group comparisons and the ROC analyses were repeated for all parameters.

Alpha risk was set to 0.05. Statistical analysis was performed with EasyMedStat (version 3.21.5; www.easymedstat.com).

## 3 Results

### 3.1 Total Population

A total of 45 individuals were initially recruited to participate in the study for a total of 90 ankles. Twelve ankles were excluded from the CLAI group due to LAS history without meeting the International Ankle Consortium's criterie for CLAI, while six were excluded because they had self‐reported feelings of instability without a history of LAS. Eight ankles were excluded from the control group because they had CLAI on the contralateral side (Supporting Information S1: Figure SC). In total, the CLAI and Control groups consisted of 32 ankles each. These 64 ankles were contributed by 42 participants. In the CLAI group, the 32 ankles came from 23 participants (14 contributed one ankle and 9 contributed both ankles). In the Control group, the 32 ankles came from 19 participants (6 contributed one ankle and 13 contributed both ankles). No participant contributed ankles to both groups. The mean number of LAS was 5.2 ± 6.9 in the CLAI group and 0 in the Control group (*p* < 0.001). Demographics, sport characteristics and ankle instability questionnaire results are presented in Table 1.

**TABLE 1:** Demographics, sport characteristics and questionnaire results.

	CLAI group (*n* = 32)	Control group (*n* = 32)	*p* values
Age (years; mean ± SD)	25.8 ± 5.5	28.2 ± 6.6	0.25
Body Mass index (kg/m^2^; mean ± SD)	23.5 ± 3.2	23.8 ± 3.1	0.69
Sex	10 women (31.2%)	12 women (37.5%)	0.79
Sport	Running (*n* = 10)	Running (*n* = 13)	0.33
	Cycling (*n* = 7)	Tennis (*n* = 4)	
	Basketball (*n* = 4)	Cycling (*n* = 3)	
	Weightlifting (*n* = 4)	Weightlifting (*n* = 2)	
	Tennis (*n* = 3)	Soccer (*n* = 2)	
	Soccer (*n* = 1)	Table tennis (*n* = 2)	
	Golf (*n* = 1)	Swimming (*n* = 2)	
	Triathlon (*n* = 1)	Dance (*n* = 2)	
	Volleyball (*n* = 1)	Basketball (*n* = 1)	
		Volleyball (*n* = 1)	
Competition/leisure	7 competition (21.9%)	4 competition (12.5%)	0.51
Sports hours/month (mean ± SD)	19.9 ± 9.8	18.1 ± 11.7	0.23
Number of LAS (mean ± SD)	5.2 ± 6.9	0	< 0.001
IdFAI score (mean ± SD)	17.2 ± 13.4	2.5 ± 2.4	< 0.001
FAAM ADLs subscale (mean ± SD)	81.3 ± 5.9	83.8 ± 0.7	< 0.001
FAAM sports subscale (mean ± SD)	30.6 ± 2.2	31.8 ± 0.5	< 0.001

Abbreviations: ADLs, Activities of Daily Living; CLAI, Chronic Lateral Ankle Instability; FAAM, Foot Ankle Ability Measurement; IdFAI, Identification of Functional Ankle Instability; LAS, Lateral Ankle Sprain; SD, Standard Deviation.

No fall or injuries were reported in this study.

### 3.2 Differentiating CLAI From Controls

**FIGURE 3:**
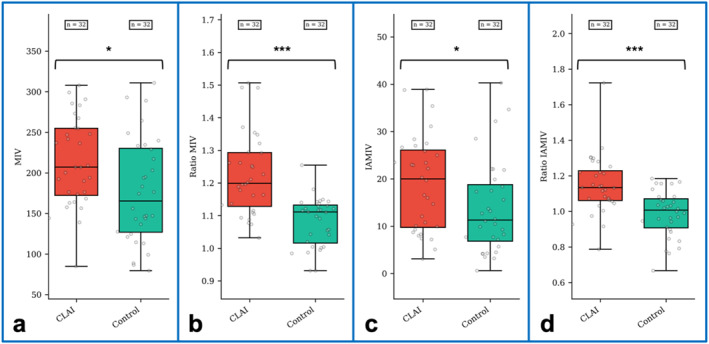
Comparisons between Chronic Lateral Ankle Instability (CLAI) and control groups; (a) Comparison of Maximum Inversion Velocity (MIV); (b) Comparison of Ratio MIV; (c) Comparison on Inversion Angle at the MIV Time (IAMIV); (d) Comparison of Ratio IAMIV * *p* < 0.05; **p < 0.01; ***p < 0.001.

In the CLAI and Control groups, the mean MIV values were 213.5°/s (SD 54.7°/s) and 177°/s (SD 64.2°/s), respectively (*p* = 0.02). The median IAMIV values were 20.0° (IQR 16.4°) and 11.3° (IQR 12.0°) in the CLAI and Control groups, respectively (*p* = 0.03). Mean Ratio MIV values were 1.22 (SD 0.13) and 1.08 (SD 0.08) in the CLAI and Control groups respectively (*p* < 0.001). Median Ratio IAMIV values were 1.13 (IQR 0.17) and 1.01 (IQR 0.16) in the CLAI and Control groups respectively (*p* < 0.001) (Figure 3).

The areas under the curve to predict CLAI were 0.68 (95% CI; [0.55; 0.81]) for MIV, 0.66 (95% CI; [0.52; 0.79]) for IAMIV, 0.83 (95% CI; [0.73; 0.93]) for Ratio MIV and 0.79 (95% CI; [0.67; 0.9]) for Ratio IAMIV. A Ratio MIV greater than 1.15 showed a 68% sensitivity and 93.8% specificity (Youden index = 0.63) and a Ratio IAMIV greater than 1.07 had a 71.9% sensitivity and 75.0% specificity (Youden index = 0.47) in identifying CLAI patients (Supporting Information S1: Figures SD–E).

In the participant‐level sensitivity analysis (one value per participant, *n* = 42), all parameters remained significant and discriminative: MIV (*p* = 0.003; AUC = 0.73), IAMIV (*p* = 0.015; AUC = 0.70), Ratio MIV (*p* < 0.001; AUC = 0.87) and Ratio IAMIV (*p* < 0.001; AUC = 0.84). The between‐group differences and discriminative ability were therefore preserved at the participant level.

### 3.3 Association With Functional CLAI

**FIGURE 4:**
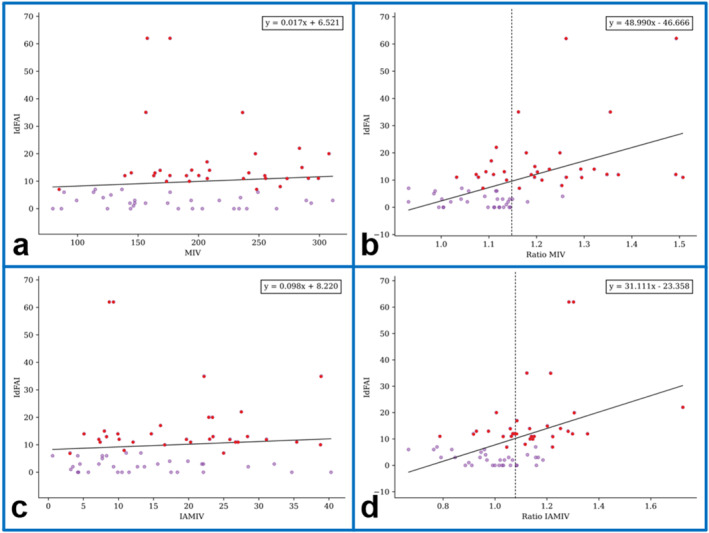
Correlation diagrams, including proposed cut‐off values (dashed lines) between the Identification of Functional Ankle Instability (IdFAI) questionnaire and: (a) Maximal Inversion Velocity (MIV); (b) Ratio MIV; (c) Inversion Angle at the MIV Time (IAMIV); (d) Ratio IAMIV. Red dots: Chronic Lateral Ankle Instability (CLAI) group. Purple dots: Control Group.

In univariate analysis, no correlation was found between IdFAI and MIV (*ρ* = 0.22; *p* = 0.08) and between IdFAI and IAMIV (*ρ* = 0.19; *p* = 0.14). A moderate positive correlation was found between IdFAI and Ratio MIV (*ρ* = 0.50; *p* < 0.0001). A low positive correlation was found between IdFAI and Ratio IAMIV (*ρ* = 0.44; *p* < 0.001) (Table 2, Figure 4).

**TABLE 2:** Univariate analysis.

	MIV	IAMIV	Ratio MIV	Ratio IAMIV
IdFAI	*ρ* = 0.22 *p* = 0.08	*ρ* = 0.19 *p* = 0.14	*ρ* = 0.5 *p* < 0.001	*ρ* = 0.44 *p* < 0.001
FAAM	*ρ* = −0.08 *p* = 0.52	*ρ* = 0.04 *p* = 0.77	*ρ* = −0.45 *p* < 0.001	*ρ* = −0.29 *p* = 0.02
ADLs Subscale				
FAAM	*ρ* = −0.05 *p* = 0.71	*ρ* = −0.06 *p* = 0.67	*ρ* = −0.43 *p* < 0.001	*ρ* = −0.20 *p* = 0.11
Sports Subscale				

Abbreviations: ADLs, Activities of Daily Living; FAAM, Foot Ankle Ability Measurement; IAMIV, Inversion angle at the MIV time; IdFAI, Identification of Functional Ankle Instability; MIV, Maximum Inversion Velocity.

### 3.4 Association With Impairments in Activities of Daily Living and Sports

No correlation was found between the FAAM ADL Subscale and MIV (*ρ* = −0.08; *p* = 0.52) and IAMIV (*ρ* = 0.04; *p* = 0.77), respectively. A low negative correlation was found between FAAM ADL Subscale and Ratio MIV (*ρ* = −0.45; *p* < 0.001). While a poor negative correlation was found between the FAAM ADL Subscale and Ratio IAMIV (*ρ* = −0.29; *p* = 0.02) (Table 2).

No correlation was found between the FAAM Sports Subscale and MIV (*ρ* = −0.05; *p* = 0.71) and IAMIV (*ρ* = −0.06; *p* = 0.67). A low negative correlation was found between the FAAM Sports Subscale and Ratio MIV (*ρ* = −0.43; *p* < 0.001). No significant correlation was found between the FAAM Sports Subscale and Ratio IAMIV (*ρ* = −0.20; *p* = 0.11) (Table 2).

## 4 Discussion

In this study, CLAI and Control subjects differed with respect to inversion velocities and inversion angles during a simulated ankle sprain caused by a novel ankle sprain simulator. A Ratio MIV greater than 1.15 showed ability in identifying CLAI subjects. Additionally, an increased Ratio MIV was associated with an increase in functional CLAI and increased levels of impairments in activities of daily living and sports.

Several studies have used sprain simulators to compare CLAI and controls [23, 24, 38]. However, this study's simulated ankle sprain is unique in that it occurred both unexpectedly and during normal walking conditions, allowing the ankle to naturally invert. The unexpectedness of the trauma is necessary to reliably induce an ankle inversion moment that more truly mimics an ankle sprain. Otherwise, if there is anticipation of the instability moment, the peroneal muscles pre‐activate and their anticipated contractions control and protect the ankle from the inversion motion [31, 39, 40]. While it is necessary for the inversion event to be unexpected, it is difficult to balance the need for an unexpected trauma with the need to avoid injury during testing. The inversion velocities observed here (213.5°/s in CLAI and 177.0°/s in controls) are consistent with the simulated‐sprain range reported by Chu et al. (114°–203°/s) and with the values reported by Simpson et al. during tilted‐surface landings (167°–187°/s) [25, 26]. Further, the values observed in this study remain well below both the proposed 300°/s safety threshold and the 632°/s recorded during a real injury event [41].

Unlike earlier diagnostic studies that relied on tilt‐platforms or drop‐landings in an anticipated or static condition, our protocol assessed the ankle during an unexpected perturbation while walking. The Myolux device was previously validated only as a rehabilitation tool [36]. To our knowledge, the present study is the first to use it as a diagnostic instrument to differentiate CLAI from controls.

While we did not experience any falls or injuries during the present study, 2 ankles slightly exceeded this previously suggested threshold (311°/s for a Control ankle and 308°/s for a CLAI ankle, Figure 4). It is reassuring that not only are these values well below the 632°/s previously reported, but also that no subjects reported ankle pain after participation in the present study. It is likely that more data on both actual sprains as well as simulated sprains is needed if a precise global threshold for injury risk is wanted. Additionally, each individual's amount of ligamentous laxity or their maximal ankle range of motion, among other risk factors, may play a role in determining each subject's risk of injury. Therefore, we recommend using parameters normalized to each individual, such as Ratio MIV, in an attempt to define a more precise threshold for individual injury risk.

The weak correlation observed for the raw values (MIV, IAMIV) and the stronger correlation for the normalised values (Ratio MIV, Ratio IAMIV) reflect the high inter‐individual variability of inversion velocity, which supports the use of individually normalised parameters. The simulator and the functional scores capture complementary information: the questionnaires reflect the patient's perception of instability, while the simulator provides an objective measure of the dynamic ankle response.

Finally, an exploratory analysis was completed to investigate the ability of Ratio MIV and Ratio IAMIV to identify CLAI patients. Ratio MIV was the better variable with a value greater than 1.15 demonstrating a 68% sensitivity and 93.8% specificity. This initial potential cutoff of 1.15 will likely be further refined with a larger cohort that includes individuals with greater impairments. A potential diagnostic measure, such as Ratio MIV, may then be used to evaluate particular patient characteristics with the goal of quantifying relative importance of the risk factors that lead to CLAI development.

The results of this study suggests that patients with CLAI can be identified and be studied safely, reliably, and repeatedly using this ankle sprain simulator. While many risk factors for the development of CLAI have been described [8, 42] a limitation of current CLAI literature is a lack of causal understanding [43]. Therefore, the question remains unanswered: why do some patients fully recover from an initial LAS while some go on to develop CLAI? This question is best answered by prospective studies that quantify CLAI in vivo, during a dynamic and unexpected episode of instability. The sprain simulator described here fits this role. It is the hope of the authors' that further research will be able to comprehensively investigate CLAI utilizing this tool that best recreates the natural physiologic state of an ankle sprain in a controlled setting.

### 4.1 Limitations

Our study has several limitations. First, only physically active individuals under the age of forty were included, which limits the extrapolation of our findings to the general population. However, physically active people are the most affected by LAS and CLAI [44, 45] and they represent a significant part of the general population. Similarly, we composed our group according to the most current recommendations of the International Ankle Consortium, which could have caused selection bias. We also utilized questionnaires (IdFAI and FAAM) to represent functional CLAI and daily life impairments. Nevertheless, these are the current reference recommendations on the subject. Second, the subjects' walking was not perfectly normal as the boots likely altered the walking pattern. Although not perfect, the authors argue that this study's protocol better reflects an actual LAS than jumping on a platform or experiencing an expected sudden movement in a static position.

Finally, some participants contributed both ankles while other participants contributed one ankle. Although a participant‐level sensitivity analysis using one averaged value per participant gave the same results, a residual within‐subject dependence cannot be fully excluded.

## 5 Conclusions

Inversion velocities caused by an ankle sprain simulator clearly differentiated CLAI from healthy controls in our study. A Ratio MIV greater than 1.15 showed ability in identifying CLAI subjects. Increasing values of Ratio MIV was associated with functional CLAI including impairments of activities of daily living and impairments in sports. This tool may be used in the future to study LAS and CLAI by safely simulating an unexpected instability moment while walking.

## Author Contributions

**Matthieu Lalevée:** data curation, formal analysis, investigation, project administration, software, writing – original draft, writing – review and editing. **Marie‐Anne Melone:** validation, methodology, investigation, resources. **Maxime l’Hermette:** methodology, project administration, validation, resources, writing – review and editing. **Julien Beldame:** validation, resources, methodology. **Cesar de Cesar Netto:** conceptualization, project administration, supervision. **Eli Schmidt:** investigation, data curation, formal analysis, writing – review and editing. **Jason Wilken:** supervision, conceptualization, methodology, resources, validation.

## Funding

The authors have nothing to report.

## Conflicts of Interest

The authors declare no conflicts of interest.

## Supporting information


Supporting Information S1

## Data Availability

The data that support the findings of this study are available from the corresponding author upon reasonable request.
