# Supplementary material for: Evaluation of Chronic Lateral Ankle Instability With an Ankle Sprain Simulator: A Controlled Study in Physically Active Subjects
Source: J Foot Ankle Res. 2026 Jul 15;19(3):e70182. doi: 10.1002/jfa2.70182 (PMC13373532; doi:10.1002/jfa2.70182)
Supplement: Supplementary file 1 — Supporting Information S1 [file JFA2-19-e70182-s001.docx]

**Supplemental Figures**


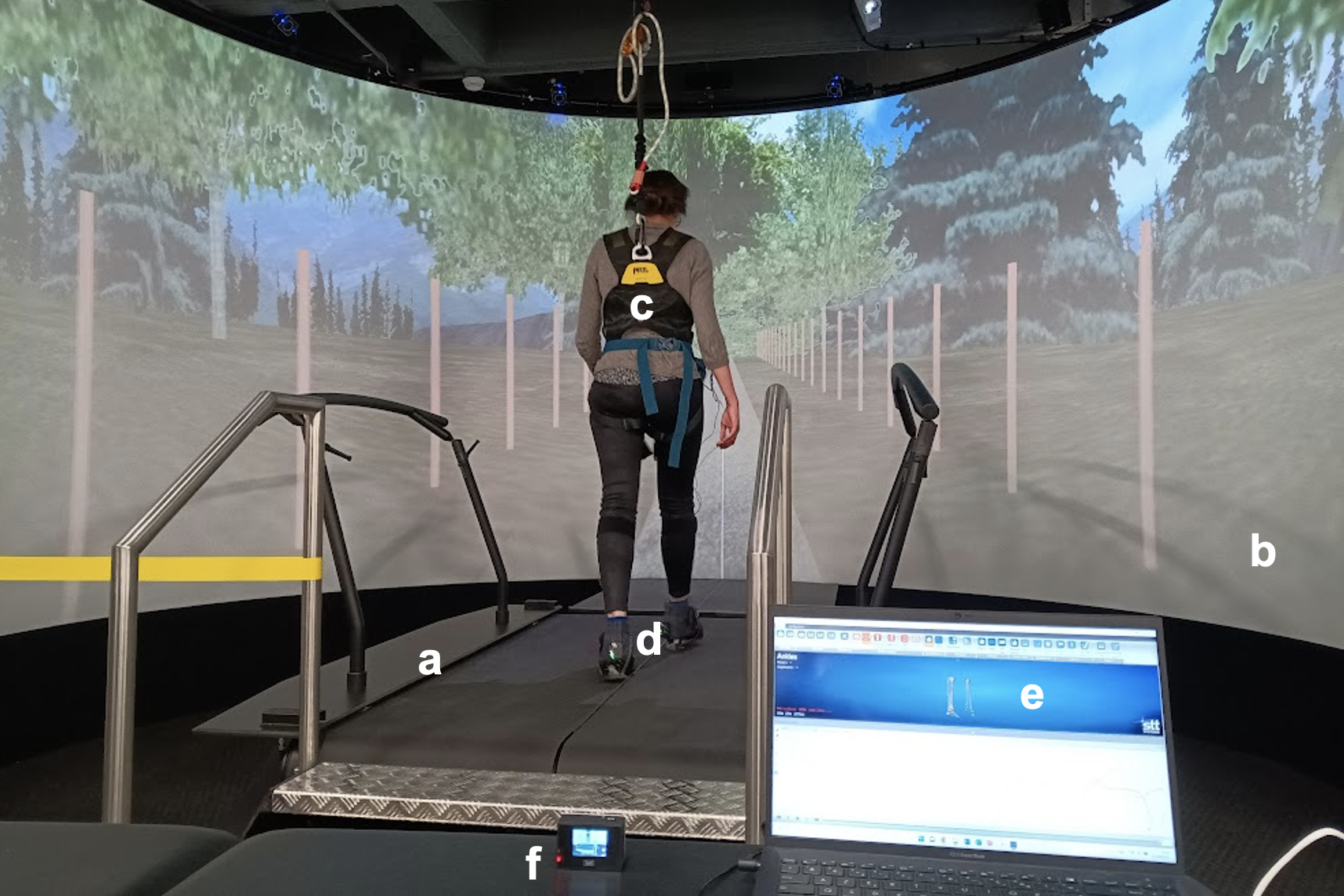


**Figure A:** Gait analysis of a subject

1. *Treadmill of the Computer Assisted Rehabilitation Environment system (CAREN, Motek^®^)*
2. *Virtual reality screen of the Computer Assisted Rehabilitation Environment system (CAREN, Motek^®^)*
3. *Security harness*
4. *Instability boots (Myolux Athletik^®^) coupled with Inertial Moment Unit sensors (Isen 3.0 STT Systems^®^)*
5. *Gait analysis software (Isen 3.0 STT Systems^®^)*
6. *Video recording*


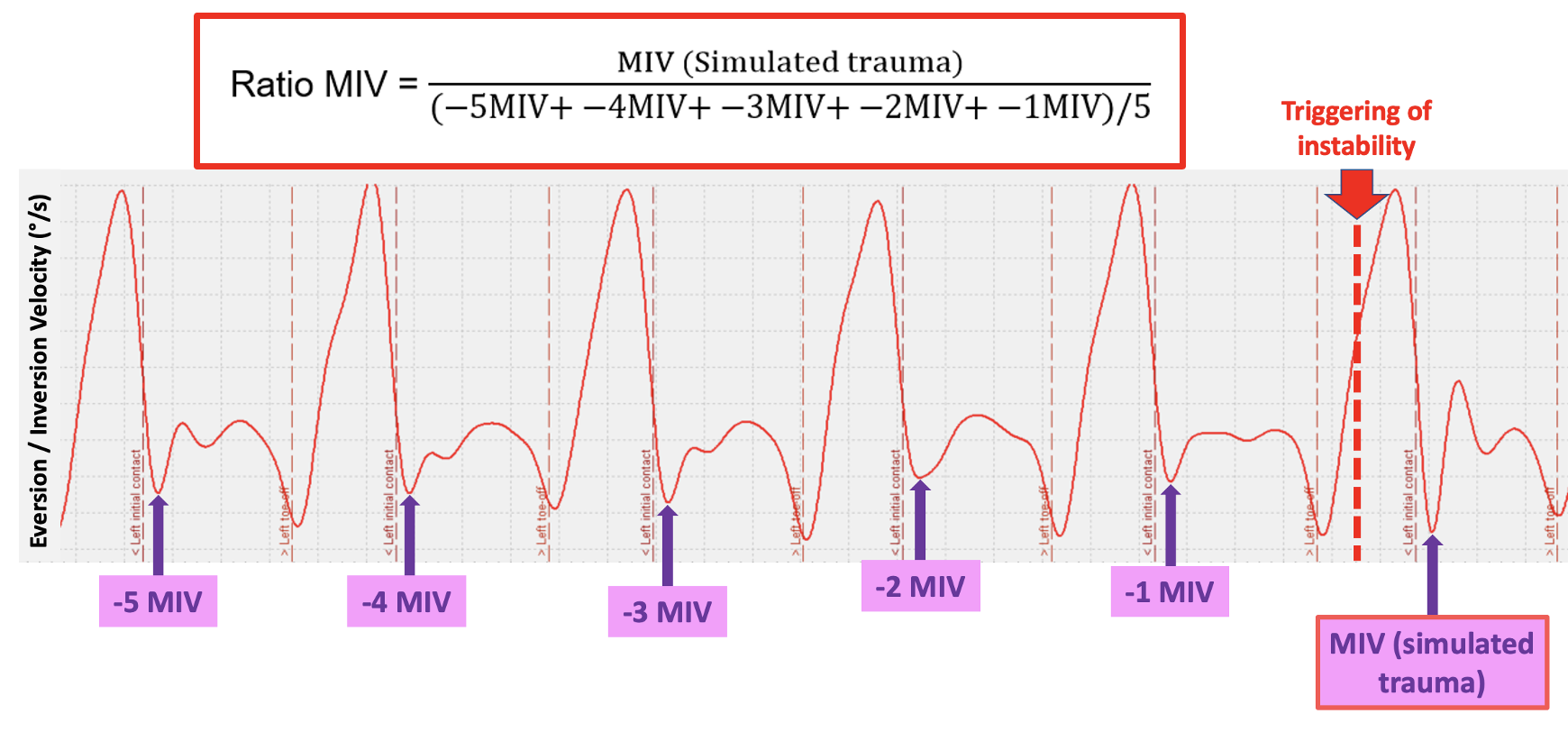


**Figure B:** Eversion/Inversion velocities during the onset of ankle instability showing the calculation of Ratio MIV

*MIV, Maximal Inversion Velocity*


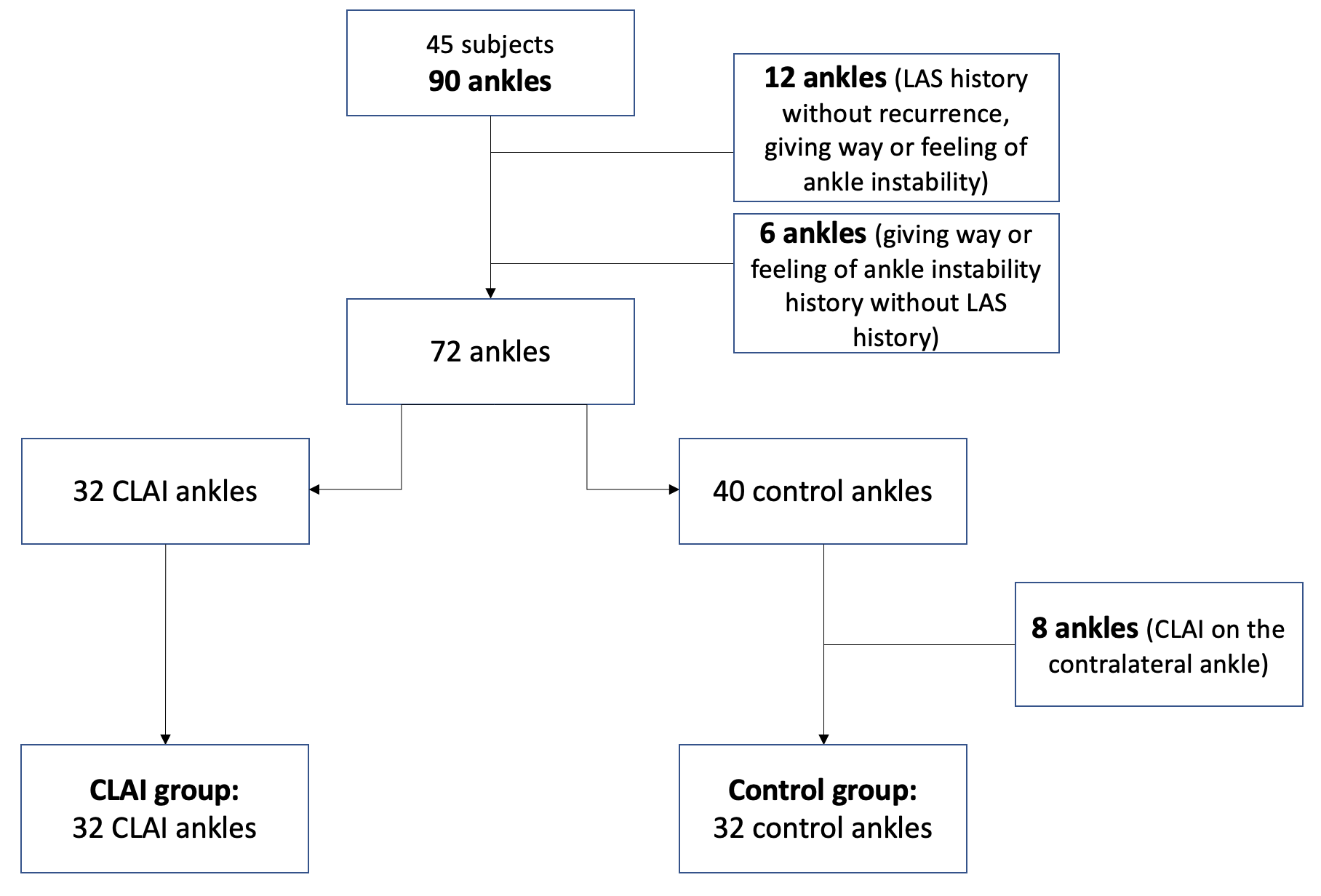


**Figure C:**  Flow Chart of Included Patients

*LAS, Lateral Ankle Sprain; CLAI, Chronic Lateral Ankle Instability*


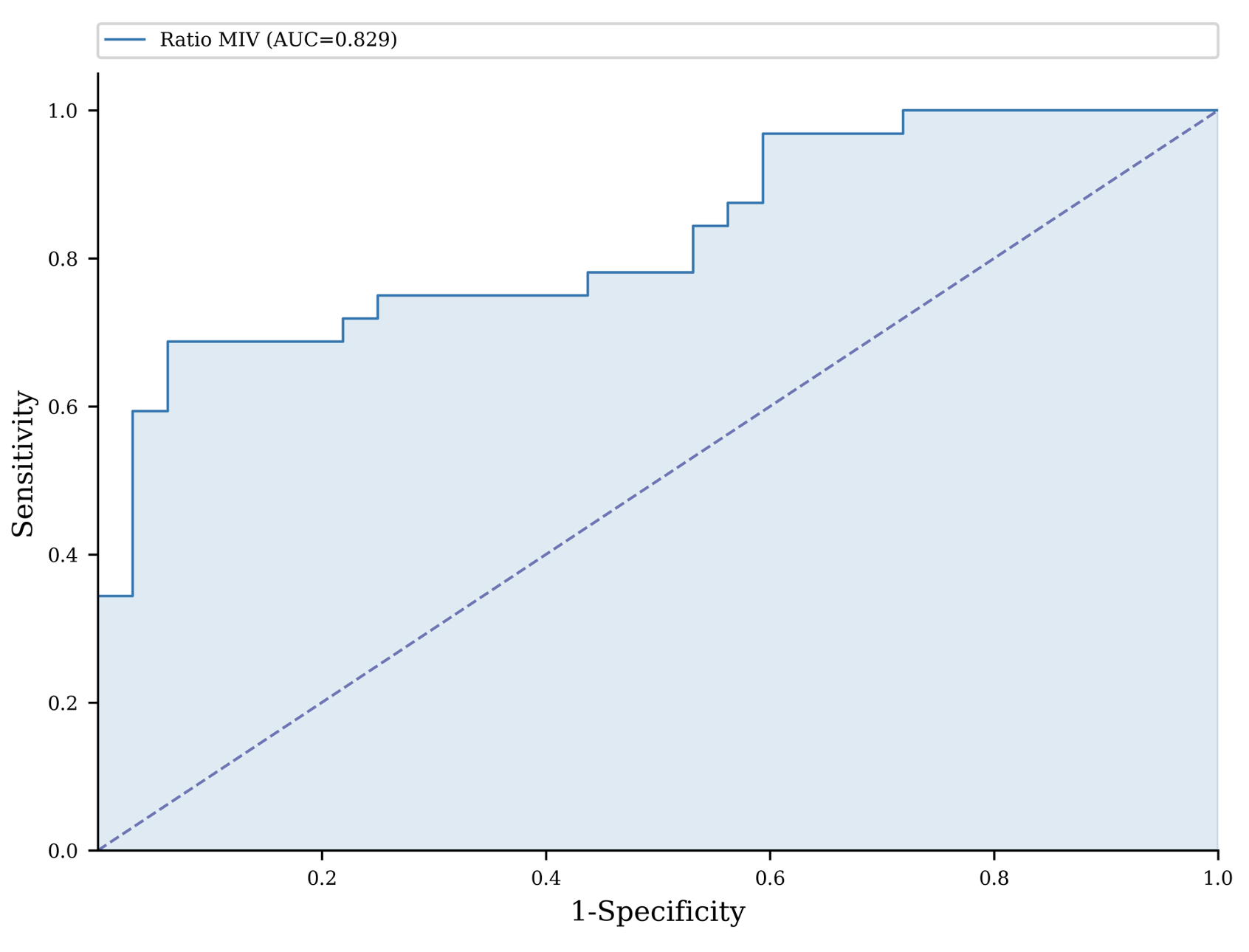


**Figure D:** Receiver operating characteristic curve for Ratio MIV (area under the curve = 0.68)

*MIV, Maximal Inversion Velocity*


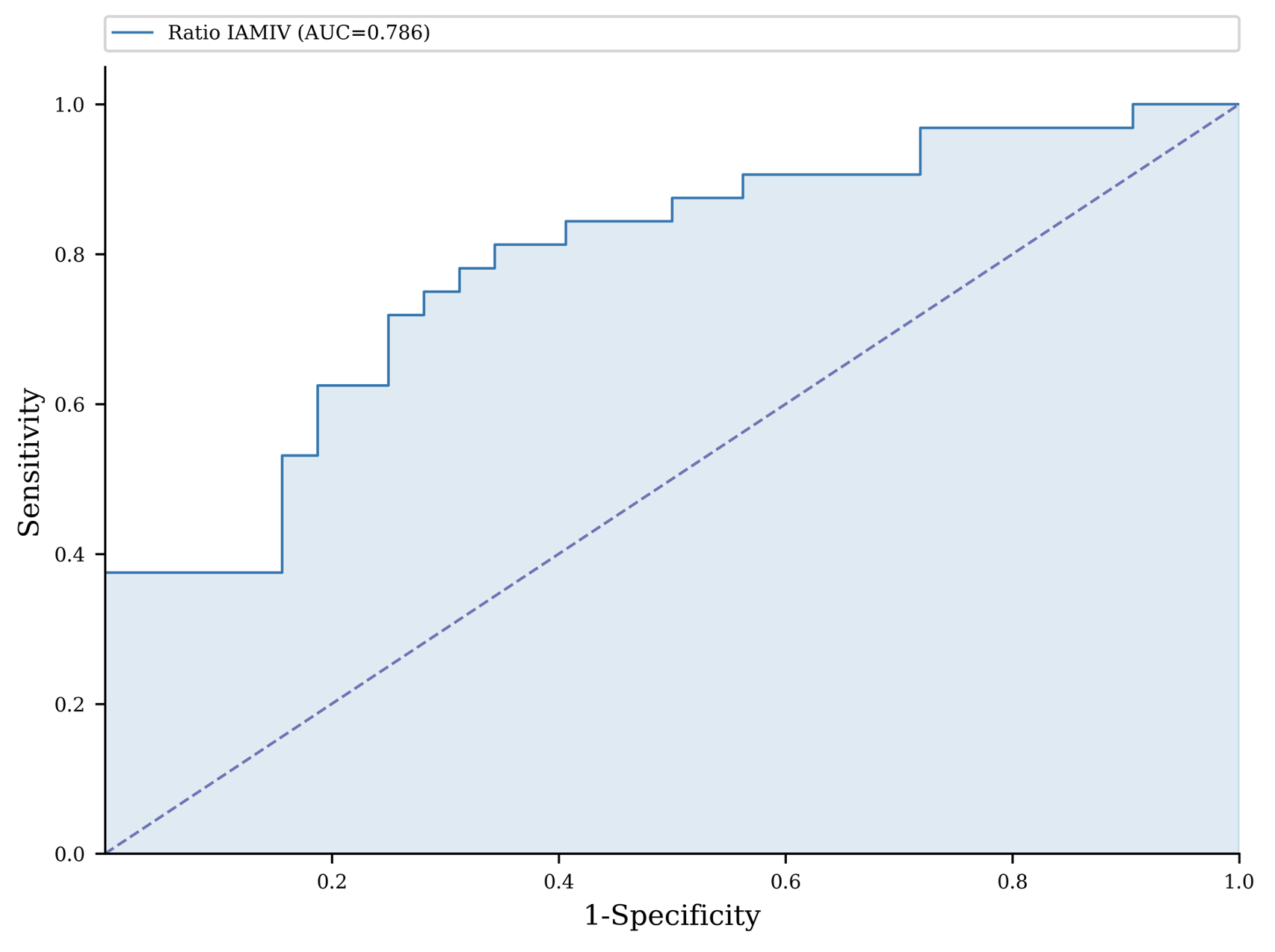


**Figure E:** Receiver operative characteristic curve for Ratio IAMIV (area under the curve = 0.79)

*IAMIV, Inversion Angle at Maximal Inversion Velocity (MIV) Time*
